# Complete Genome Sequence of *Latilactobacillus* sp. Strain WDN19, a High-d-Aspartate-Producing Lactic Acid Bacterium Isolated from a Japanese Pickle

**DOI:** 10.1128/MRA.00568-21

**Published:** 2021-08-26

**Authors:** Kengo Kajitani, Shouji Takahashi

**Affiliations:** a Department of Bioengineering, Nagaoka University of Technology, Nagaoka, Niigata, Japan; University of Arizona

## Abstract

We report here the complete genome sequence of *Latilactobacillus* sp. strain WDN19, isolated from a Japanese pickle. This strain can produce a large amount of d-aspartate in the culture broth. The genome consists of a circular chromosome (1,967,462 bp; GC content, 41.88%) and a circular plasmid (66,648 bp; GC content, 35.08%).

## ANNOUNCEMENT

The amino acid d-aspartate (d-Asp) is an important intermediate for synthetic penicillin (1). Some lactic acid bacteria (LAB) produce d-Asp as a component of cell wall peptidoglycan (2). LAB strains with greater d-Asp production would therefore be valuable for industrial d-Asp production. We isolated a novel high-d-Asp-producing strain, *Latilactobacillus* sp. strain WDN19, from a Japanese pickle purchased at a market in Niigata Prefecture, Japan (3). Here, we report the complete genome sequence of strain WDN19. This genome sequence will provide an avenue for the biotechnological manipulation of strain WDN19 for increased production of d-Asp.

Strain WDN19 was isolated from a Japanese pickle as described previously (3) and stored as glycerol stock at −80°C. To prepare the genomic DNA of strain WDN19, a single colony grown on MRS agar medium was transferred to 14 ml MRS medium (BD Difco, Sparks, MD, USA), statically cultured until the early stationary phase, approximately 8 h at 30°C, and pelleted. Genomic DNA was isolated from the pelleted cells using the Genomic-tip 100/G kit (Qiagen, Hilden, Germany) according to the manufacturer’s protocol with modifications for Gram-positive bacteria. A SMRTbell library was prepared and sequenced using a single-molecule real-time (SMRT) cell in a PacBio RS II sequencer at Macrogen Corp. Japan (Tokyo, Japan) for long-read sequencing. A total of 1.21 Gbp sequence data (coverage, 597×) in 121,749 filtered subreads was obtained. The average length and *N*_50_ value of the subreads were 9,972 and 13,416 bp, respectively. The PacBio data were assembled using the Hierarchical Genome Assembly Process 3 (HGAP3) in the PacBio SMRT Analysis version 2.3.0 platform (4). Gene prediction and annotation of the generated contigs were performed using the DDBJ Fast Annotation and Submission Tool (DFAST) version 1.2.13 (https://dfast.ddbj.nig.ac.jp/). A BLASTn search of the 16S rRNA gene sequence of strain WDN19 was performed against the NCBI rRNA/ITS databases (https://blast.ncbi.nlm.nih.gov/Blast.cgi). The average nucleotide identity (ANI) was calculated using JSpeciesWS (5). Default parameters were used for all software unless otherwise specified.

The complete genome of strain WDN19 consists of a single circular chromosome 1,967,462 bp long with a GC content of 41.88% and a single circular plasmid 66,648 bp long with a GC content of 35.08%. DFAST predicted that the chromosome contains 2,055 protein-coding sequences (CDSs), 64 tRNA genes, and 18 rRNA genes and the plasmid sequence contains 70 CDSs and 1 tRNA gene. The 16S rRNA gene sequence of strain WDN19 had the highest similarity to that of Latilactobacillus curvatus DSM 20019 (=NBRC 15884^T^) (GenBank accession number AB626051), with 99.86% identity. Furthermore, ANI analysis showed that the chromosome sequence of strain WDN19 has the highest similarity to that of strain DSM 20019 (GCA_001435495.1), with 98.5% identity, which is above the threshold value of 95 to 96% ANI for species delineation (6), suggesting that strain WDN19 is a member of *L. curvatus*.

### Data availability.

The complete genome sequence of strain WDN19 has been deposited in the DDBJ/EMBL/GenBank databases under accession numbers AP024685 for the chromosome and AP024686 for the plasmid. The raw reads were deposited under BioProject accession number PRJDB11678, BioSample accession number SAMD00323104, and DDBJ Sequence Read Archive (DRA) accession number DRA012183.
